# Complement C3 Deficiency Attenuates Chronic Hypoxia-Induced Pulmonary Hypertension in Mice

**DOI:** 10.1371/journal.pone.0028578

**Published:** 2011-12-14

**Authors:** Eileen M. Bauer, Han Zheng, Suzy Comhair, Serpil Erzurum, Timothy R. Billiar, Philip M. Bauer

**Affiliations:** 1 Department of Surgery, University of Pittsburgh School of Medicine, Pittsburgh, Pennsylvania, United States of America; 2 Department of Pharmacology and Chemical Biology, University of Pittsburgh School of Medicine, Pittsburgh, Pennsylvania, United States of America; 3 Vascular Medicine Institute, University of Pittsburgh School of Medicine, Pittsburgh, Pennsylvania, United States of America; 4 Department of Pathobiology, Lerner Research Institute, Cleveland Clinic, Cleveland, Ohio, United States of America; 5 Respiratory Institute, Cleveland Clinic, Cleveland, Ohio, United States of America; University of Illinois at Chicago, United States of America

## Abstract

**Background:**

Evidence suggests a role of both innate and adaptive immunity in the development of pulmonary arterial hypertension. The complement system is a key sentry of the innate immune system and bridges innate and adaptive immunity. To date there are no studies addressing a role for the complement system in pulmonary arterial hypertension.

**Methodology/Principal Findings:**

Immunofluorescent staining revealed significant C3d deposition in lung sections from IPAH patients and C57Bl6/J wild-type mice exposed to three weeks of chronic hypoxia to induce pulmonary hypertension. Right ventricular systolic pressure and right ventricular hypertrophy were increased in hypoxic vs. normoxic wild-type mice, which were attenuated in C3−/− hypoxic mice. Likewise, pulmonary vascular remodeling was attenuated in the C3−/− mice compared to wild-type mice as determined by the number of muscularized peripheral arterioles and morphometric analysis of vessel wall thickness. The loss of C3 attenuated the increase in interleukin-6 and intracellular adhesion molecule-1 expression in response to chronic hypoxia, but not endothelin-1 levels. In wild-type mice, but not C3−/− mice, chronic hypoxia led to platelet activation as assessed by bleeding time, and flow cytometry of platelets to determine cell surface P-selectin expression. In addition, tissue factor expression and fibrin deposition were increased in the lungs of WT mice in response to chronic hypoxia. These pro-thrombotic effects of hypoxia were abrogated in C3−/− mice.

**Conclusions:**

Herein, we provide compelling genetic evidence that the complement system plays a pathophysiologic role in the development of PAH in mice, promoting pulmonary vascular remodeling and a pro-thrombotic phenotype. In addition we demonstrate C3d deposition in IPAH patients suggesting that complement activation plays a role in the development of PAH in humans.

## Introduction

Pulmonary arterial hypertension (PAH) is a progressive disease characterized by increased pulmonary vascular resistance and pulmonary arterial pressure leading to right heart failure [1]. The pathogenesis of PAH is complex involving pulmonary vasoconstriction, remodeling of the pulmonary vascular wall, and *in situ* thrombosis [2]. It is becoming increasingly recognized that immune system activation and inflammation play important roles in the pathogenesis of PAH [3].

The complement system is a key sentry of innate immunity acting as a first line of defense against injurious stimuli and invading pathogens [4]. It may be activated by the classical, alternative or lectin pathways. All three pathways converge at the level of C3 cleavage and activation leading to the production of opsonins (C3b), the membrane attack complex (C5b-9) [5], and anaphylatoxins (C3a and C5a). The anaphylatoxins are particularly interesting as potential effectors in PAH because they recruit inflammatory cells, cause degranulation of mast cells, increase vascular permeability and stimulate pulmonary vascular smooth muscle contraction [6], [7], [8]. In addition, complement components C3 and C4a have been implicated as biomarkers of idiopathic pulmonary hypertension [9], [10]. To date, however, there are no studies exploring a role for complement activation in PAH. In this study we utilized C3−/− mice to explore the role of complement in chronic hypoxia (CH)-induced PAH in mice.

## Methods

### Ethics Statement

Human tissue and cell samples were obtained in compliance with Cleveland Clinic and University of Pittsburgh institutional review board guidelines as previously described [11], [12], [13], [14]. Animal studies were approved by the University of Pittsburgh Institutional Animal Care and Use Committee (University of Pittsburgh Animal Assurance # A3187-01).

### Mice

C57Bl/6J (stock #000664) and C3 −/− mice (stock #003641) were purchased from Jackson Laboratories. C3 −/− mice are reported by the Jackson Laboratories to be backcrossed to the C57BL/6J background for 7 generations. All experiments were performed on age-matched male mice between 8–10 weeks old.

### Materials

Complement component C3d antibody (AF2655), complement component C5a antibody (AF2150) and recombinant mouse complement component C5a (2150-C5) were from R&D Systems. Recombinant human complement C3a (204881) and recombinant human complement C5a (234397) were from Calbiochem. Tissue factor antibody (SATF-IG) was from Affinity Biologicals.

### Chronic hypoxia-induced pulmonary hypertension

Pulmonary hypertension was induced by housing mice under chronic hypoxic conditions (FiO_2_ = 0.10, normobaric) for three weeks with age matched mice in normoxia serving as control.

### Measurement of right ventricular systolic pressure (RVSP)

Mice were anesthetized with sodium pentobarbital (50 mg/kg i.p.) and ventilated via tracheotomy with room air (175 breaths/min, 175 µl tidal volume). Body temperature was monitored and regulated with a rectal probe and heating pad. Right ventricular systolic pressure (RVSP) was determined by placing a 1F solid state pressure transducing catheter (Millar Instruments Inc., Houston, TX) directly into the RV. Data were acquired using a PowerLab data acquisition system and LabChart Pro software (AD Instruments).

### RV hypertrophy (RVH)

RVH was determined by the ratio of the weight of the RV to the left ventricle plus septum (Fulton index) as previously described [15].

### Pulmonary vascular remodeling

Lung sections were stained against smooth muscle alpha actin antibody (1∶100, DAKO) after deparaffinization and antigen retrieval. Pulmonary vascular remodeling was assessed by counting the number of partially and fully muscularized peripheral arterioles (35–100 mm) per high power field (200× total magnification). For each mouse, more than 20 high power fields were analyzed in multiple lung sections. Wall thickness of muscularized vessels was determined by measuring the thickness at 4 points on pulmonary arterioles using the Java-based image processing program: Image J (NIH).

### Cultured Cells

Human pulmonary artery smooth muscle cells (hPASMC) [16] and human aortic smooth muscle cells (hASMC) [17] were isolated as previously described. hPASMC were grown in DMEM/F12 media supplemented with 10% FBS and penicillin/streptomycin and were maintained at 37°C and 5% CO_2_. hASMC were maintained in SMC growth medium (Cell Applications, San Diego, CA). Cells were used between passage 4–9.

### Cell Proliferation

Proliferation of hASMC and hPASMC was determined by measuring [^3^H]-incorporation as previously described [18]. Briefly, cells were serum starved for 24 h in 12-well plates and treated with either 10 or 100 nM Human C3a or C5a with or without PDGF (10 ng/ml,Sigma P4056) for 24 h. During the last 16 hrs 0.2 µCi [^3^H]thymidine was added. After the incubation period cells were washed twice with ice-cold PBS, and 1 ml of ice-cold 10% trichloroacetic acid (Sigma T0699) was added to each well for a 30-min incubation at 4°C, after which each well was washed with 1 ml of ice-cold 10% trichloroacetic acid. To each well 0.5 ml of 0.4 N NaOH, 0.1% (wt/vol) SDS was added, and the plates were incubated for 1 h at room temperature. The contents of each well were then transferred to 7 ml scintillation vials containing 4.5 ml of Pico-Fluor-15 scintillation mixture (ICN) and counted in a liquid scintillation spectrometer.

### Bleeding Time

Mice were anesthetized with isoflurane and a cut was made 3 mm from the tip of the tail. After transection, the tail was placed in a beaker filled with 37°C phosphate buffered saline, and bleeding time was recorded. After bleeding succession another 30 sec was waited for possible rebleeding. Bleeding was stopped at 10 min and all tails were cauterized.

### Immunohistochemistry

After sacrifice, mice were perfused intracardially with phosphate buffered saline to clear the vasculature of blood. After perfusing the lungs via the trachea with 4% paraformaldehyde the trachea was tied off, the lungs excised, and kept for an additional 24 hrs in paraformaldehyde at 4°C. The tissue was paraffin embedded and sectioned (5 µm) by the University of Pittsburgh Research Histology Laboratory. Sections were warmed for 60 min at 56°C followed by deparaffinization in Xylene (3 times 3 min) and rehydrated to PBS (100% Ethanol 2×3 min, 90% Ethanol 1×3 min, 70% Ethanol 1×3 min and PBS 1×3 min). All slides were treated for antigen retrieval in citrate buffer followed by blocking and staining for either smooth muscle-α-actin (1∶100 DAKO), fibrin (Nordic Immunology, polyclonal goat), Von Willebrand Factor (Santa Cruz, polyclonal rabbit), C3d (R&D Systems, goat) following manufacturer's recommendations and appropriate HRP- or fluorescently-labeled secondary antibodies. For morphometric analysis, the Vectastin Elite ABC DAB kit was used to visualize smooth muscle actin staining and images captured by light microspcopy. Fluorescently-labeled antibodies were detected by confocal microscopy using an Olympus Fluoview 1000. For all immunohistochemical staining a no primary control was used to confirm the specificity of the staining.

### Quantification of C3d and Fibrin Deposition

Percent area of C3d and fibrin deposition was quantified using computer-assisted image analysis (Adobe Photoshop 5.0, NIH Image J) with the observer blinded as to tissue source.

### ELISA

ELISA for endothelin-1 and intracellular adhesion molecule-1 were performed as per the manufacturer's instructions (R&D Systems).

### Flow Cytometry

Mouse blood was obtained via cardiac puncture at the time of sacrifice using syringes containing 100 ul of citrate phosphate dextrose solution and spun at room temp for 15 min at 1000 rpm to obtain platelet rich plasma (PRP). PRP was transferred and labeled with PE-anti-mouse-CD41 and FITC-anti-mouse-CD62P (BD bioscience) antibodies following the manufacturer's protocol for “staining platelets for activation”. PE-IgG1-k or FITC IgG1-λ were used as isotype control. Cells were analyzed by FACS (Guava-easy-cyte *HT) using Guava Express Pro 8.1 software, gating for CD41 positive cells (platelet marker).

### Immunoprecipitation

C5a was immunoprecipitated from plasma or lung using anti-C5a antibody conjugated to protein A/G Dynal beads (Invitrogen) per the manufacturer's instructions [19].

### Western blot

Tissue homogenates were separated by SDS–PAGE and transferred to nitrocellulose membranes. Membranes were blocked in TBST (Tris buffered saline, 0.1% Tween 20), 5% non-fat dry milk for 30 min, followed by incubation in primary antibody. Membranes were washed in TBST before incubation for 1 h with horseradish peroxidase-conjugated secondary antibodies. Membranes were washed and developed using enhanced chemiluminescence substrate (Pierce). The intensity of the bands was quantified using Image J software (rsbweb.nih.gov/ij/).

### Real time PCR

RNA was isolated after homogenization of snap frozen lungs using the RNeasy Mini Kit (Qiagen). RNA was reverse transcribed using random hexamers and reverse transcriptase enzyme (Applied Biosystems). Taqman primer/probe mix for interleukin-6, and β2 microglobulin were from Applied Biosystems. IL-6 expression was normalized to β2-microglobulin.

### Statistical Analysis

Data were analyzed by Student's T-test when comparing two groups or by one-way ANOVA and Tukey's post-hoc test when comparing 3 or more groups. *P*<0.05 was considered significant.

## Results

**Figure 1:**
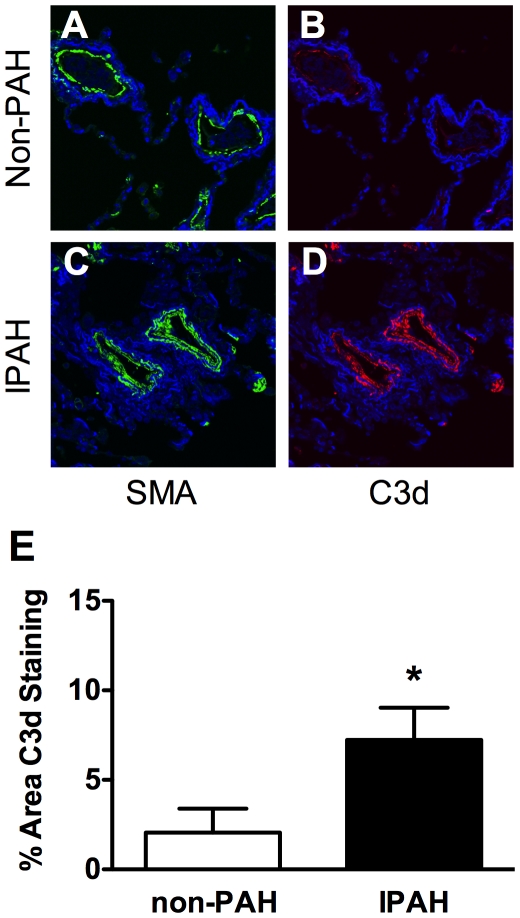
C3d deposition in human PAH. (A–D) Lung sections from non-PAH or IPAH patients (n = 3) were stained with α-C3d and α-SMA antibody and counterstained with DAPI to detect nuclei. Images shown are representative (E) Quantification of C3d staining in non-PAH and IPAH patients. Bars represent the mean ± SD (n = 4). *P<0.05.

**Figure 2:**
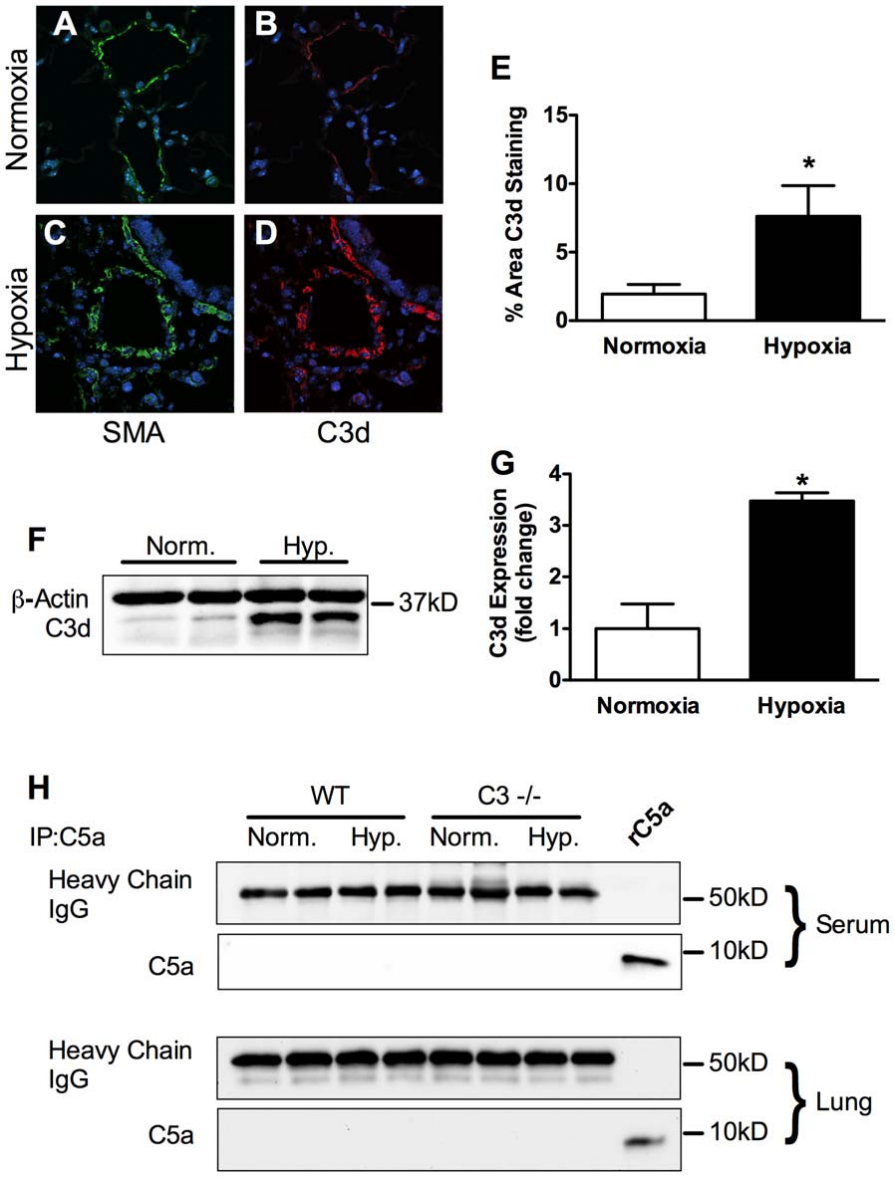
C3d deposition in chronic hypoxia-induced PH in mice. (A–D) Lungs from normoxic and hypoxic C57Bl/6J mice were stained with α-C3d and α-SMA antibody and counterstained with DAPI to detect nuclei(n = 4). (E) Quantification of C3d staining in WT vs. C3−/− mice in normoxia and hypoxia. Bars represent the mean ± SD (n = 4). *P<0.05. (F) Representative Western blot for C3d in normoxic vs. hypoxic C57Bl/6J mice. (G) Quantification of Western blots for C3d in normoxic vs. hypoxic mice. Bars represent the mean ± SD (n = 4). *P<0.05. (H) C5a was immunoprecipitated from lung or plasma of normoxic and hypoxic C57Bl/6J mice and analyzed by Western blot (rC5a = recombinant mouse C5a).

Complement deposition was assessed in lung sections of IPAH or control patients by immunofluorescent staining with anti-C3d antibody. We detected significant C3d deposition in the vascular wall of IPAH patients whereas it was nearly undetectable in control lungs (Fig. 1A–D). Likewise, C57BL/6J mice exposed to 3-weeks CH, but not normoxic controls, showed significant C3d deposition in the vascular wall (Fig. 2A–D). Quantification of C3d staining reveals that C3d deposition was increased approximately 3.5-fold in humans (Fig. 1E) and 3.9-fold in mouse (Fig. 2E). Western blot analysis of lung from normoxic and hypoxic C57BL/6J mice showed a similar 3.5 fold increase in C3d in the hypoxic mouse lung (Fig. 2F–G). Immunoprecipitation of C5a from plasma or lung followed by Western blot analysis for C5a failed to detect the C5 cleavage product suggesting that activation of complement in chronic hypoxia is limited to C3 (Fig. 2H). Recombinant mouse C5a served as a positive control.

**Figure 3:**
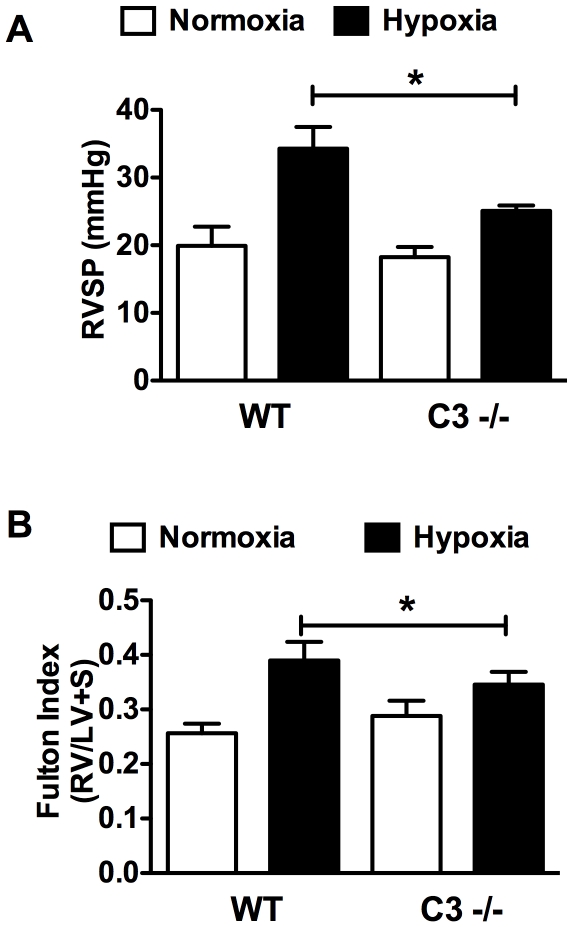
Genetic deletion of C3 attenuates CH-induced PAH in mice. WT or C3−/− mice were exposed to CH to induce PAH. After 3 weeks (A) RVSP and (B) RVH (Fulton index), were determined. Bars represent the mean ± SD (n = 8–12). *P<0.05.

We next compared the effect of CH on WT vs. C3−/− mice. CH elicited a significant increase in RVSP in WT mice (19.9 mmHg, normoxia vs. 34.3 mmHg, hypoxia) that was attenuated in the C3−/− mice (18.3 mmHg, normoxia vs. 25.1 mm Hg, hypoxia) (Fig. 3A). Likewise, RVH, a normal sequelae of increased RVSP, was attenuated in C3−/− hypoxic mice as determined by the fulton index (0.390, WT vs. .346, C3−/−) (Fig. 3B).

**Figure 4:**
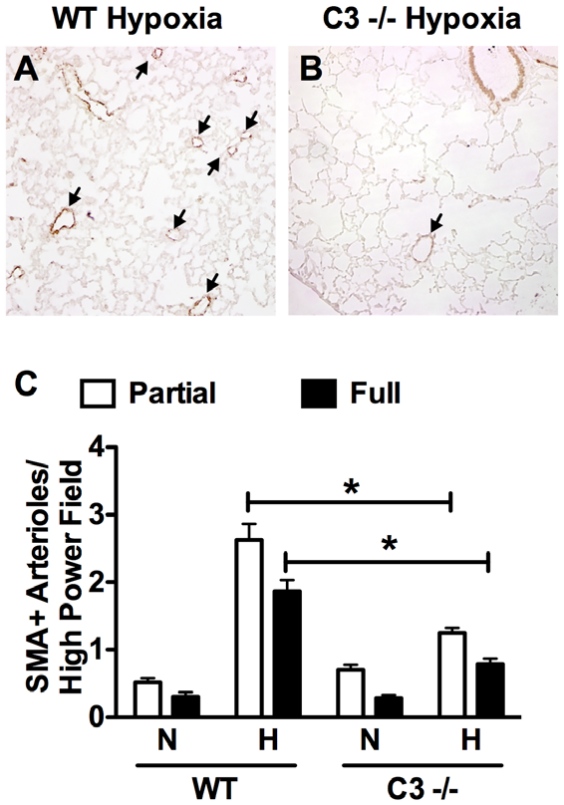
Loss of C3 attenuates muscularization of pulmonary arterioles in response to CH. (A–B) Representative photomicrographs of lungs stained against SMA from (A) hypoxic WT and (B) C3−/− mice. Arrows indicate muscularized arterioles. (C) Quantification of the number of partially and fully muscularized arterioles (<100 µm) per high power field (200× total magnification). Bars represent the mean ± SD (n = 4). *P<0.05.

**Figure 5:**
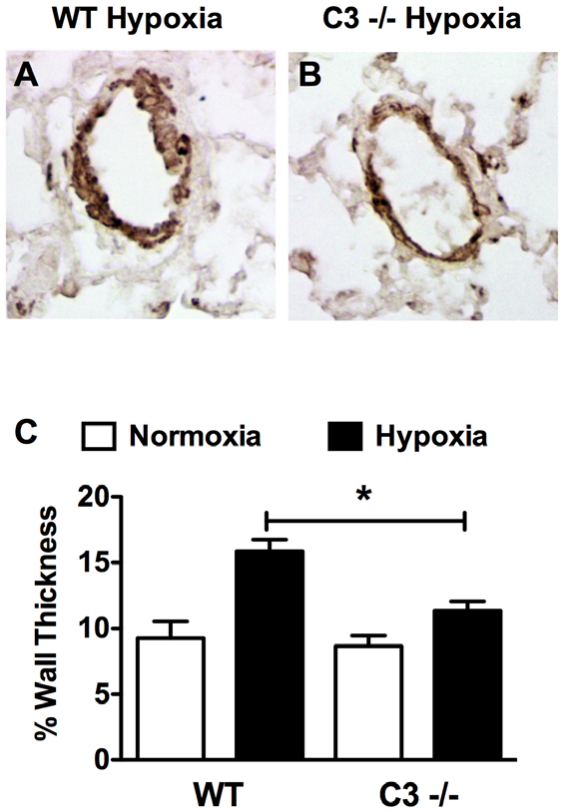
Loss of C3 attenuates vascular wall thickening in response to CH. (A–B) Representative photomicrographs of muscularized arterioles from (A) hypoxic WT and (B) hypoxic C3−/− mice. (C) Quantification of the % wall thickness of peripheral arterioles (<100 µm). Bars represent the mean ± SD (n = 4). *P<0.05.

**Figure 6:**
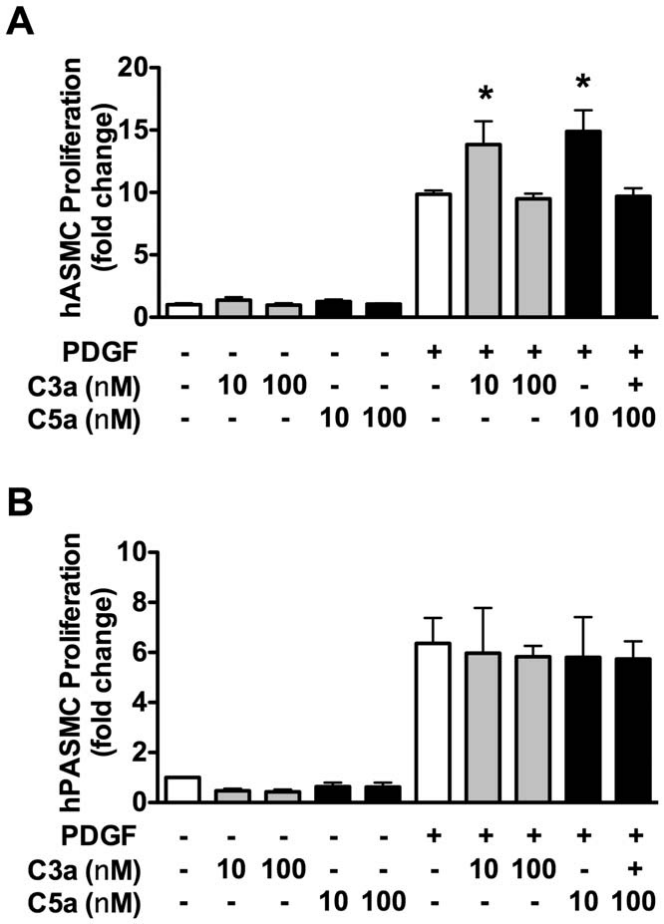
Complement C3a and C5a do not promote hPASMC proliferation. (**A**) **hASMC and** (**B**) hPASMC were treated with C3a or C5a with or without concurrent PDGF (10 ng/ml) stimulation and assessed for proliferation. Bars represent mean ± SD of four individual experiments. *P<0.05 vs. PDGF. n.s. = not significant.

Increased peripheral vascular resistance leading to elevated RVSP and RVH is in part caused by pulmonary vascular remodeling characterized by the muscularization and increased wall thickness of small (>100 µm) non-muscular arterioles. C3−/− hypoxic mice showed significantly less pulmonary vascular remodeling compared to WT hypoxic mice. SMA staining of the lung revealed significantly less muscularization of small peripheral arterioles in hypoxic C3−/− mice vs. WT mice (Fig. 4). Likewise, morphometric analysis of small peripheral arterioles revealed significantly less thickening of the vessel wall in C3−/− mice vs. WT mice (Fig. 5). Previous studies show that C3a, a cleavage product of C3, stimulates aortic smooth muscle cells [20] and genetic deletion of C3a receptor or C5a receptor attenuates neointimal hyperplasia and vascular smooth muscle cell proliferation after arterial injury. To confirm this finding we stimulated human aortic smooth muscle cells with C3a or C5a in the presence or absence of platelet-derived growth factor. In our hands C3a and C5a alone had no effect on hASMC proliferation, but enhanced PDGF-stimulated hASMC proliferation at 10 nm. Interestingly, a higher concentration of C3a or C5a (100 nM) had no effect on PDGF-stimulated hASMC (Fig. 6A). In order to determine whether C3a or C5a has a similar effect on vascular smooth muscle cells in the pulmonary vasculature, hPASMC were exposed to varying concentrations of C3a or C5a ± PDGF. Neither C3a nor C5a had any effect on hPASMC proliferation alone or in the presence of PDGF (Fig. 6B).

**Figure 7:**
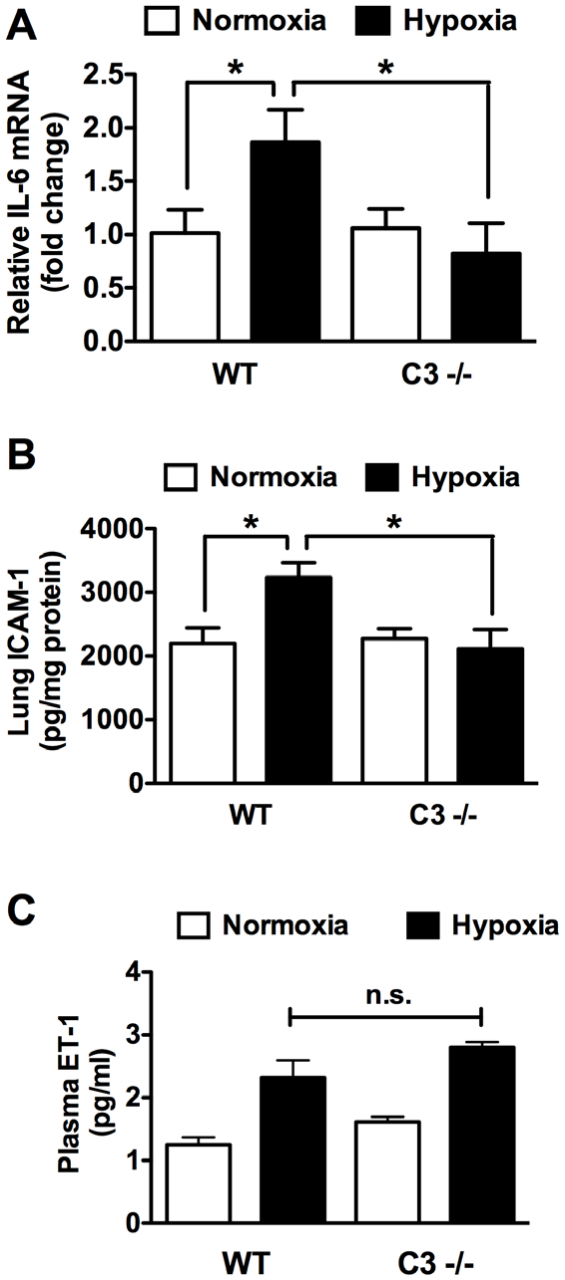
Markers of Inflammation and endothelial dysfunction in WT vs. C3 −/− mice. (A) IL-6 mRNA was measured by quantitative rtPCR in RNA prepared from normoxic or hypoxic WT and C3−/− lungs. IL-6 levels were normalized to the house keeping gene β2-microglobulin. (B) ICAM-1 was quantified by ELISA in lung homogenates from normoxic or hypoxic WT or C3−/− mice. (C) ET-1 was quantified by ELISA in plasma from normoxic or hypoxic WT or C3−/− mice. Bars represent mean ± SD (n = 4) for A–C. *P<0.05.

Endothelial dysfunction and inflammation contribute to the development of PH and are promoted by complement activation. Among inflammatory cytokines, interleukin-6 (IL-6) has recently been shown to play a prominent role in the development of pulmonary hypertension. In WT mice CH induced an approximate 2-fold increase in IL-6 mRNA at three weeks that was abrogated in C3 −/− mice (Fig. 7A). In terms of endothelial dysfunction, adhesion molecules and vasoconstrictors play important roles in PH. We observed increases in lung intracellular adhesion molecule 1 (ICAM-1) and plasma endothelin-1 (ET-1) in WT mice exposed to CH. Interestingly, while the increase in ICAM-1 was abrogated in C3 −/− mice the increase in ET-1 was not (Fig. 7B–C).

**Figure 8:**
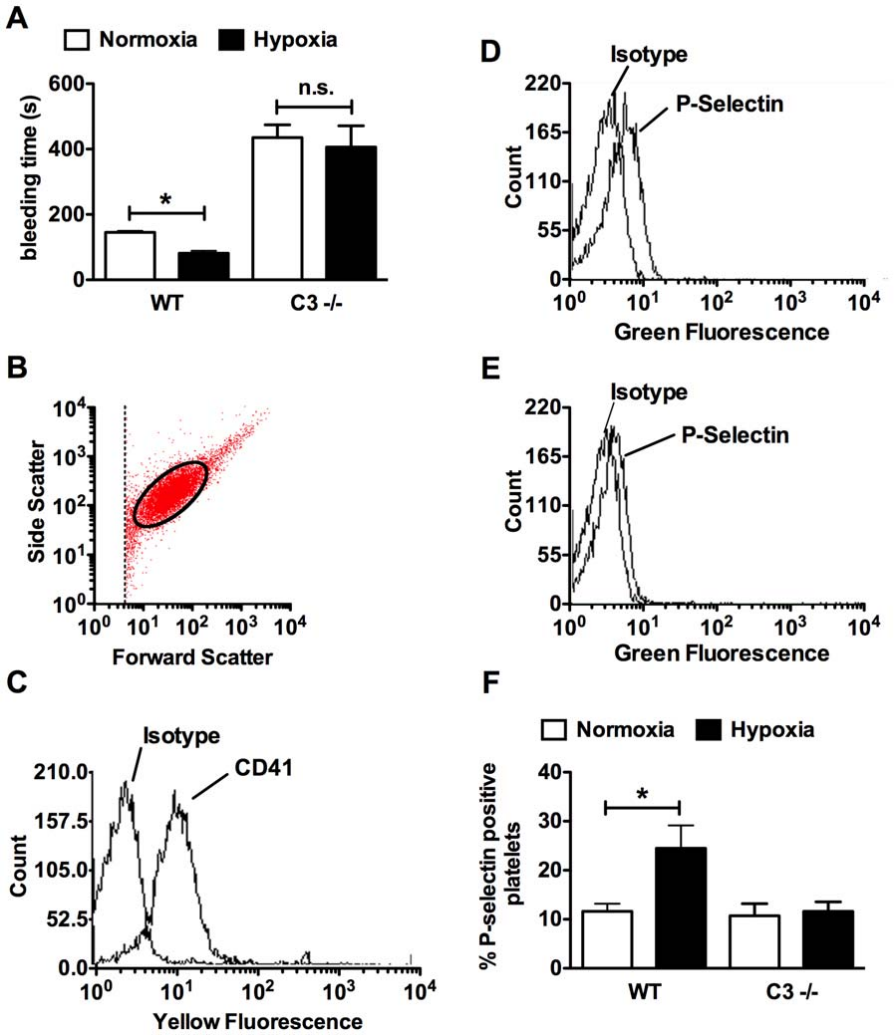
Loss of C3 prevents platelet activation caused by CH. (A) Bleeding time of WT and C3−/− mice exposed to normoxia or CH (n = 5–7). (B) Scatter plot showing platelet population in platelet rich plasma. All experiments were similarly gated to the area encircled. (C) Flow cytometry histogram demonstrating that the gated cell population is positive for the platelet marker CD41. (D–E) Representative flow cytometry histograms of platelets from (D) hypoxic WT or (E) hypoxic C3−/− mice stained with P-selectin antibody or isotype control. (F) Percent P-selectin positive platelets in PRP isolated from normoxic and hypoxic WT or C3−/− mice (n = 6). Bars represent mean ± SD. *P<0.05; n.s. = not significant.

The complement system interacts with the coagulation cascade and C3 −/− mice exhibit abnormal platelet activation. Therefore, we were interested in determining whether the loss of C3 would protect against platelet activation caused by CH. In normoxia bleeding time, a reflection of platelet activation, was significantly prolonged in C3 −/− mice as previously described [21]. In WT mice, CH led to a significant decrease in bleeding time compared to normoxic mice (Fig. 8A). In contrast, CH had no effect on bleeding time in C3−/− mice. In concordance with decreased bleeding time, the fraction of P-selectin (a marker of activated platelets) positive platelets in hypoxic WT mice, but not C3−/− mice, was significantly increased compared to normoxic control as determined by flow cytometry (Fig. 8B–F).

**Figure 9:**
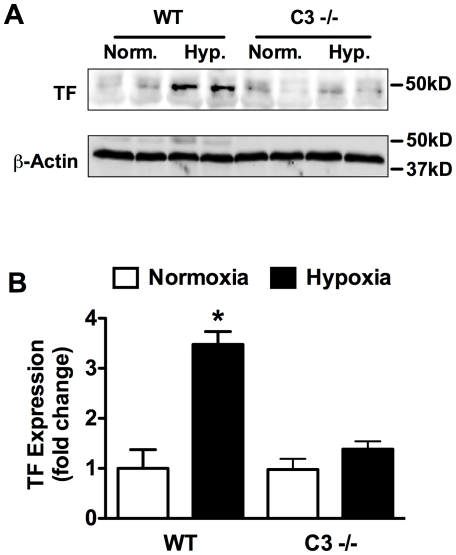
Loss of C3 prevents hypoxia-induced TF upregulation. (A) Representative Western blot analysis of normoxic or hypoxic WT and C3−/− lungs for TF expression. (B) Densitometric anlysis of Western blots from (A). Bars represent the mean ± SD (n = 4 animals per group). *P<0.05.

**Figure 10:**
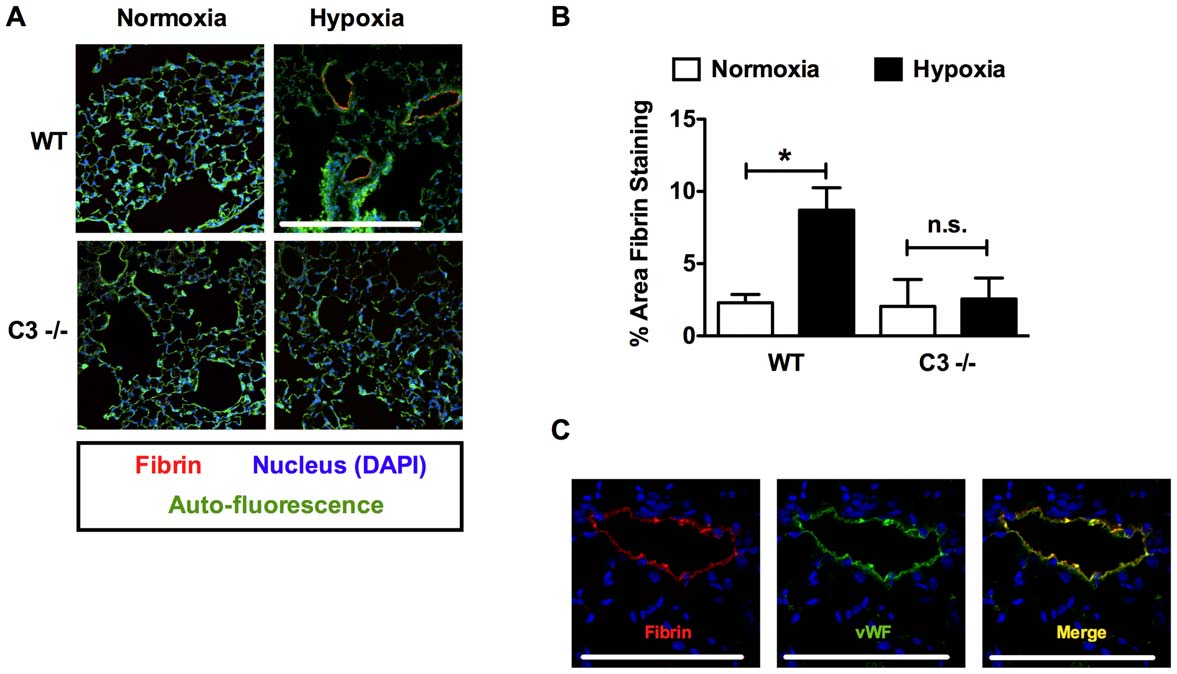
Decreased fibrin deposition in hypoxic C3−/− mice. (A) Representative images of lung sections from normoxic or hypoxic WT or C3 −/− mice stained for fibrin(ogen) and nuclei (DAPI). Scale bar represents 150 µm. (B) % Area fibrin staining per total lung area (n = 4) in hypoxic or normoxic WT or C3−/− mice. (C) Representative photomicrograph of WT hypoxic mouse lung showing colocalization (yellow to orange) of Fibrin (green) and vWF (red). Scale bar represents 50 µm. (A, B, E). Bars represent the mean ± SD. *P<0.05; n.s. = not significant.

Previous studies demonstrate that CH in mice results in increased TF expression and enhanced fibrin deposition [22], [23]. Therefore, we further explored the interaction of the complement system with the coagulation cascade by examining TF expression and fibrin deposition in WT vs. C3−/− mice. In response to 3-weeks of CH, TF expression was significantly increased in WT but not C3 −/− mice (Fig. 9A,B). Likewise, CH increased fibrin deposition in WT mice but had no effect on fibrin deposition in C3−/− mice (Fig. 10A,B). Fibrin colocalized with vWF confirming that fibrin deposition was primarily within the vessel lumen (Fig. 10C).

## Discussion

The complement system is an evolutionarily ancient and highly complex biological system playing a major role in body defense as part of the innate and adaptive immune systems. The complement system consists of three activation pathways that merge at the proteolytic cleavage of C3, the nexus of the complement system. Herein, we provide for the first time genetic evidence that the complement system contributes to the development of PAH in mice. Genetic deletion of C3 caused significant attenuation of PAH in the mouse model of CH-induced PAH. This was associated with attenuated pulmonary vascular remodeling and reversal of the pro-coagulant phenotype induced by CH exposure. Furthermore, we document the deposition of C3d, a stable C3 cleavage product, in the pulmonary vasculature of humans and mice with PAH. While genetic deficiency may lead to chronic phenotypic changes which may be the true pathogenetic factors, our data strongly support a role for C3 in the pathogenesis of PH.

Activation of C3 also leads to downstream cleavage of C5 and C5 cleavage has been shown to take place in the absence of C3 by alternative mechanisms [19]. Our results suggest that C5 does not play an active role in chronic hypoxia-induced PH since we were unable to detect the C5 cleavage product C5a in plasma or lung tissue. That being said, we can not rule out the possibility that C5 cleavage takes place at earlier time points and then dissipates by three weeks.

The loss of C3 led to decreased pulmonary vascular remodeling in response to CH. The complement component C3a stimulates the proliferation of systemic vascular smooth muscle cells [20] and genetic deletion of C3a receptor or C5a receptor attenuates neointimal hyperplasia and vascular smooth muscle cell proliferation after arterial injury [24]. Our studies confirm a role for C3a and C5a in enhancing PDGF-stimulated human aortic smooth muscle cell proliferation. In contrast, C3a or C5a had no effect on the proliferation of hPASMC. These data suggest that either different complement components are responsible for promoting hPASMC proliferation or that complement activation contributes to pulmonary vascular remodeling by indirect means. Cobra venom factor, which causes rapid activation of C3 (and then depletion), elicits acute pulmonary vasoconstriction leading to a transient increase in pulmonary arterial pressure [25]. Our data suggest more chronic complement activation, which could contribute to sustained vasoconstriction and pulmonary vascular remodeling. In addition, TF as well as chronic exposure to fibrin stimulates hPASMC proliferation [26]. Finally, ICAM-1 is a ligand for lymphocyte function-associated antigen 1 (LFA-1), a receptor found on leukocytes [27]. Activated leukocytes bind to ECs via ICAM-1/LFA-1 allowing them to transmigrate into tissues [28]. Also C3a and C5a are potent chemoattractants promoting the recruitment of leukocytes. This suggests an additional mechanism by which complement may promote pulmonary vascular remodeling.

IL-6 has recently been shown to play a prominent role in the development of pulmonary hypertension. Overexpression of IL-6 promotes PH in mice, where as IL-6 knockout mice are protected from hypoxia-induced PH [29], [30]. In addition, elevated serum IL-6 concentrations have been reported in patients with idiopathic PH or PH associated with inflammatory diseases such as scleroderma and lupus [31], [32], [33]. Our data suggest that complement activation may play a role in stimulating IL-6 production in pulmonary hypertension since IL-6 expression was increased in hypoxic WT mice but not in C3 −/− mice.

In contrast to IL-6 we found that the ET-1 was increased in response to CH in both WT and C3 −/− mice. ET-1 is a potent vasoconstrictor and is a well established contributor to human PAH as evidenced by the fact that the ET-1 receptor antagonist, Bosentan, is currently used to treat PAH patients [34]. Clearly more studies need to be performed to determine whether complement inhibition might be a therapeutic avenue for treating PH. However, the fact that loss of C3 attenuates PH without affecting ET-1 levels leads to the intriguing possibility that complement inhibition might make an effective combination therapy with Bosentan.

Complement contributes to coagulation by augmenting inflammation, promoting the TF coagulation pathway, activating platelets, increasing TF expression, and modifying the activity of mast cells and basophils [35]. C3 −/− mice also exhibit defective platelet activation in response to the thrombin receptor agonist PAR4 peptide, but not collagen or ADP, suggesting a role for complement in thrombin activated platelet aggregation. Interestingly, pulmonary arterial hypertensive patients have increased platelet membrane expression of PAR1 and PAR-mediated surface exposure of P-selectin which may represent increased propensity to thrombosis [36]. Experimental models have also implicated platelet abnormalities in the thrombotic tendency of PAH. In the mouse model of hypoxia-induced PAH a small number of *in situ* vascular thrombi are found in the pulmonary vasculature [37], [38] and the development of monocrotaline-induced PAH in the rat is attenuated by inducing thrombocytopenia [39]. There are only a few clinical studies of platelet function and activation in patients with PAH. A case report described thrombocytosis in association with increased pulmonary vascular-specific fibrin generation and platelet activation in a patient with PAH [40]. Moreover, urinary metabolites of thromboxane A2 (TxA2) are increased in PAH vs control subjects [41]. This is consistent with significant platelet activation since TxA2 production is predominantly from platelets.

In our experiments CH led to decreased bleeding time and increased surface expression of P-selectin (CD62P) on platelets in WT mice providing evidence of platelet activation in PAH. In contrast, C3−/− mice had prolonged bleeding time in normoxia as previously described, and hypoxia had no effect on bleeding time or surface P-selectin expression in these mice. These data suggest that complement activation contributes to platelet activation in CH-induced pulmonary hypertension.

In addition to platelet activation, CH led to increased TF expression and fibrin deposition in WT mice but not C3−/− mice. TF is a procoagulant protein that triggers the extrinsic coagulation cascade leading to the generation of thrombin and conversion of fibrinogen to fibrin. Independent of its procoagulant activity TF also stimulates vascular cell migration and proliferation [42], [43]. Multiple lines of evidence suggest that the TF pathway may be involved in the pathogenesis of PAH. In humans, increased pulmonary expression of TF and an increase in TF-bearing microparticles have been observed [44], [45]. TF is also expressed in pulmonary plexiform lesions in humans and in a rat model of severe pulmonary hypertension [46], [47]. Additionally, TF expression is strongly induced by hypoxia, promoting pulmonary fibrin deposition and pulmonary *in situ* thrombosis [22], [23], [48], [49].

While there is little thrombosis per se in the mouse model of CH-induced PAH, increased fibrin deposition in WT but not C3 −/− mice demonstrate that, in CH, complement contributes to a pro-thrombotic environment. In addition, like TF, fibrin enhances hPASMC proliferation suggesting that fibrin deposition may play a role in pulmonary vascular remodeling [26]. It is also interesting that activation of the coagulation cascade leads to activation of the complement system via cleavage of C3 and C5 [50]. This suggests the intriguing possibility that in PAH there exists a cycle of complement and coagulation system activation, each promoting the other.

In conclusion, our data provide the first evidence for a direct role of the complement system in the development of pulmonary arterial hypertension. These seminal findings have the potential to open up new areas of PAH research as well as novel therapeutic avenues in the treatment of this deadly disease.

## References

[1] D'Alonzo GE, Barst RJ, Ayres SM, Bergofsky EH, Brundage BH (1991). Survival in patients with primary pulmonary hypertension. Results from a national prospective registry.. Ann Intern Med.

[2] Humbert M, Morrell NW, Archer SL, Stenmark KR, MacLean MR (2004). Cellular and molecular pathobiology of pulmonary arterial hypertension.. J Am Coll Cardiol.

[3] Mathew R (2010). Inflammation and pulmonary hypertension.. Cardiol Rev.

[4] Markiewski MM, Lambris JD (2007). The role of complement in inflammatory diseases from behind the scenes into the spotlight.. Am J Pathol.

[5] Pangburn MK, Rawal N (2002). Structure and function of complement C5 convertase enzymes.. Biochem Soc Trans.

[6] Bjork J, Hugli TE, Smedegard G (1985). Microvascular effects of anaphylatoxins C3a and C5a.. J Immunol.

[7] Morel DR, Zapol WM, Thomas SJ, Kitain EM, Robinson DR (1987). C5a and thromboxane generation associated with pulmonary vaso- and broncho-constriction during protamine reversal of heparin.. Anesthesiology.

[8] Morganroth ML, Schoeneich SO, Till GO, Ward PA, Horvath SJ (1990). C3a57–77, a C-terminal peptide, causes thromboxane-dependent pulmonary vascular constriction in isolated perfused rat lungs.. Am Rev Respir Dis.

[9] Abdul-Salam VB, Paul GA, Ali JO, Gibbs SR, Rahman D (2006). Identification of plasma protein biomarkers associated with idiopathic pulmonary arterial hypertension.. Proteomics.

[10] Zhang J, Zhang Y, Li N, Liu Z, Xiong C (2009). Potential diagnostic biomarkers in serum of idiopathic pulmonary arterial hypertension.. Respir Med.

[11] Aldred MA, Comhair SA, Varella-Garcia M, Asosingh K, Xu W (2010). Somatic chromosome abnormalities in the lungs of patients with pulmonary arterial hypertension.. Am J Respir Crit Care Med.

[12] Fijalkowska I, Xu W, Comhair SA, Janocha AJ, Mavrakis LA (2010). Hypoxia inducible-factor1alpha regulates the metabolic shift of pulmonary hypertensive endothelial cells.. Am J Pathol.

[13] Masri FA, Xu W, Comhair SA, Asosingh K, Koo M (2007). Hyperproliferative apoptosis-resistant endothelial cells in idiopathic pulmonary arterial hypertension.. Am J Physiol Lung Cell Mol Physiol.

[14] Xu W, Kaneko FT, Zheng S, Comhair SA, Janocha AJ (2004). Increased arginase II and decreased NO synthesis in endothelial cells of patients with pulmonary arterial hypertension.. FASEB J.

[15] Zuckerbraun BS, Shiva S, Ifedigbo E, Mathier MA, Mollen KP (2010). Nitrite potently inhibits hypoxic and inflammatory pulmonary arterial hypertension and smooth muscle proliferation via xanthine oxidoreductase-dependent nitric oxide generation.. Circulation.

[16] Aytekin M, Comhair SA, de la Motte C, Bandyopadhyay SK, Farver CF (2008). High levels of hyaluronan in idiopathic pulmonary arterial hypertension.. Am J Physiol Lung Cell Mol Physiol.

[17] Phillippi JA, Klyachko EA, Kenny JPt, Eskay MA, Gorman RC (2009). Basal and oxidative stress-induced expression of metallothionein is decreased in ascending aortic aneurysms of bicuspid aortic valve patients.. Circulation.

[18] Bauer PM, Buga GM, Ignarro LJ (2001). Role of p42/p44 mitogen-activated-protein kinase and p21waf1/cip1 in the regulation of vascular smooth muscle cell proliferation by nitric oxide.. Proc Natl Acad Sci U S A.

[19] Huber-Lang M, Sarma JV, Zetoune FS, Rittirsch D, Neff TA (2006). Generation of C5a in the absence of C3: a new complement activation pathway.. Nat Med.

[20] Verdeguer F, Castro C, Kubicek M, Pla D, Vila-Caballer M (2007). Complement regulation in murine and human hypercholesterolemia and role in the control of macrophage and smooth muscle cell proliferation.. Cardiovasc Res.

[21] Gushiken FC, Han H, Li J, Rumbaut RE, Afshar-Kharghan V (2009). Abnormal platelet function in C3-deficient mice.. J Thromb Haemost.

[22] Lawson CA, Yan SD, Yan SF, Liao H, Zhou YS (1997). Monocytes and tissue factor promote thrombosis in a murine model of oxygen deprivation.. J Clin Invest.

[23] Yan SF, Zou YS, Gao Y, Zhai C, Mackman N (1998). Tissue factor transcription driven by Egr-1 is a critical mechanism of murine pulmonary fibrin deposition in hypoxia.. Proc Natl Acad Sci U S A.

[24] Sakuma M, Morooka T, Wang Y, Shi C, Croce K (2010). The intrinsic complement regulator decay-accelerating factor modulates the biological response to vascular injury.. Arterioscler Thromb Vasc Biol.

[25] Cheung AK, Parker CJ, Wilcox L (1989). Effects of two types of cobra venom factor on porcine complement activation and pulmonary artery pressure.. Clin Exp Immunol.

[26] Firth AL, Yau J, White A, Chiles PG, Marsh JJ (2009). Chronic exposure to fibrin and fibrinogen differentially regulates intracellular Ca2+ in human pulmonary arterial smooth muscle and endothelial cells.. Am J Physiol Lung Cell Mol Physiol.

[27] Rothlein R, Springer TA (1986). The requirement for lymphocyte function-associated antigen 1 in homotypic leukocyte adhesion stimulated by phorbol ester.. J Exp Med.

[28] Yang L, Froio RM, Sciuto TE, Dvorak AM, Alon R (2005). ICAM-1 regulates neutrophil adhesion and transcellular migration of TNF-alpha-activated vascular endothelium under flow.. Blood.

[29] Steiner MK, Syrkina OL, Kolliputi N, Mark EJ, Hales CA (2009). Interleukin-6 overexpression induces pulmonary hypertension.. Circ Res.

[30] Savale L, Tu L, Rideau D, Izziki M, Maitre B (2009). Impact of interleukin-6 on hypoxia-induced pulmonary hypertension and lung inflammation in mice.. Respir Res.

[31] Soon E, Holmes AM, Treacy CM, Doughty NJ, Southgate L (2010). Elevated levels of inflammatory cytokines predict survival in idiopathic and familial pulmonary arterial hypertension.. Circulation.

[32] Pendergrass SA, Hayes E, Farina G, Lemaire R, Farber HW (2010). Limited systemic sclerosis patients with pulmonary arterial hypertension show biomarkers of inflammation and vascular injury.. PLoS One.

[33] Nishimaki T, Aotsuka S, Kondo H, Yamamoto K, Takasaki Y (1999). Immunological analysis of pulmonary hypertension in connective tissue diseases.. J Rheumatol.

[34] Rubin LJ, Badesch DB, Barst RJ, Galie N, Black CM (2002). Bosentan therapy for pulmonary arterial hypertension.. N Engl J Med.

[35] Markiewski MM, Nilsson B, Ekdahl KN, Mollnes TE, Lambris JD (2007). Complement and coagulation: strangers or partners in crime?. Trends Immunol.

[36] Maeda NY, Carvalho JH, Otake AH, Mesquita SM, Bydlowski SP (2010). Platelet protease-activated receptor 1 and membrane expression of P-selectin in pulmonary arterial hypertension.. Thromb Res.

[37] Cathcart MC, Tamosiuniene R, Chen G, Neilan TG, Bradford A (2008). Cyclooxygenase-2-linked attenuation of hypoxia-induced pulmonary hypertension and intravascular thrombosis.. J Pharmacol Exp Ther.

[38] White TA, Witt TA, Pan S, Mueske CS, Kleppe LS (2009). Tissue Factor Pathway Inhibitor Overexpression Inhibits Hypoxia-induced Pulmonary Hypertension.. Am J Respir Cell Mol Biol.

[39] Ganey PE, Sprugel KH, White SM, Wagner JG, Roth RA (1988). Pulmonary hypertension due to monocrotaline pyrrole is reduced by moderate thrombocytopenia.. Am J Physiol.

[40] Rostagno C, Prisco D, Abbate R, Poggesi L (1991). Pulmonary hypertension associated with long-standing thrombocytosis.. Chest.

[41] Christman BW, McPherson CD, Newman JH, King GA, Bernard GR (1992). An imbalance between the excretion of thromboxane and prostacyclin metabolites in pulmonary hypertension.. N Engl J Med.

[42] Cirillo P, Cali G, Golino P, Calabro P, Forte L (2004). Tissue factor binding of activated factor VII triggers smooth muscle cell proliferation via extracellular signal-regulated kinase activation.. Circulation.

[43] Sato Y, Asada Y, Marutsuka K, Hatakeyama K, Sumiyoshi A (1996). Tissue factor induces migration of cultured aortic smooth muscle cells.. Thromb Haemost.

[44] Altman R, Scazziota A, Rouvier J, Gurfinkel E, Favaloro R (1996). Coagulation and fibrinolytic parameters in patients with pulmonary hypertension.. Clin Cardiol.

[45] Bakouboula B, Morel O, Faure A, Zobairi F, Jesel L (2008). Procoagulant membrane microparticles correlate with the severity of pulmonary arterial hypertension.. Am J Respir Crit Care Med.

[46] White RJ, Galaria II, Harvey J, Blaxall BC, Cool CD (2005). Tissue factor is induced in a rodent model of severe pulmonary hypertension characterized by neointimal lesions typical of human disease.. Chest.

[47] White RJ, Meoli DF, Swarthout RF, Kallop DY, Galaria II (2007). Plexiform-like lesions and increased tissue factor expression in a rat model of severe pulmonary arterial hypertension.. Am J Physiol Lung Cell Mol Physiol.

[48] Yan SF, Mackman N, Kisiel W, Stern DM, Pinsky DJ (1999). Hypoxia/Hypoxemia-Induced activation of the procoagulant pathways and the pathogenesis of ischemia-associated thrombosis.. Arterioscler Thromb Vasc Biol.

[49] Yan SF, Pinsky DJ, Stern DM (2000). A pathway leading to hypoxia-induced vascular fibrin deposition.. Semin Thromb Hemost.

[50] Amara U, Flierl MA, Rittirsch D, Klos A, Chen H Molecular intercommunication between the complement and coagulation systems.. J Immunol.

